# How does job insecurity cause unethical pro-organizational behavior? The mediating role of impression management motivation and the moderating role of organizational identification

**DOI:** 10.3389/fpsyg.2022.941650

**Published:** 2022-09-23

**Authors:** Lin Xu, Ting Wen, Jigan Wang

**Affiliations:** ^1^School of Management, Nanjing University of Posts and Telecommunications, Nanjing, China; ^2^School of Business Administration, Nanjing University of Finance and Economics, Nanjing, China; ^3^Business School, Hohai University, Nanjing, China

**Keywords:** quantitative job insecurity, qualitative job insecurity, unethical pro-organizational behavior, impression management motivation, organizational identification

## Abstract

This study aims to examine the effect of quantitative and qualitative job insecurity on unethical pro-organizational behavior (UPB), focusing on the mediating effect of impression management motivation and the moderating effect of organizational identification. A two-wave questionnaire survey is conducted, and data from 254 employees of Chinese enterprises are used to test the research hypotheses. Empirical results show that: (1) Quantitative job insecurity has a significant positive effect on UPB, while positive effect of qualitative job insecurity on UPB is insignificant. (2) Quantitative job insecurity positively affects impression management motivation and increases UPB. Although the direct effect of qualitative job insecurity on UPB is insignificant, it positively affects UPB through impression management motivation. (3) Organizational identification plays a positive moderation role in the relationship between impression management motivation and employees’ UPB, that is, high-degree organizational identification leads to a strong effect of impression management motivation on UPB; furthermore, organizational identification moderates the mediating role of impression management motivation in the relationships between quantitative, qualitative job insecurity, and UPB, such that the effect is strong when organizational identification is high, rather than low. This study compares the effect of quantitative and qualitative job insecurity on employees’ UPB, reveals that impression management motivation is the key mechanism of quantitative and qualitative job insecurity affecting UPB, and points out the moderating effect of organizational identification, which offers implications for organizational management practices.

## Introduction

With a series of business scandals (e.g., the Siemens bribery scandal and the Volkswagen emission scandal) coming to light, unethical pro-organizational behavior (UPB) has become a popular topic of academic attention. UPB is defined as “actions that are intended to promote the effective functioning of the organization or its members (e.g., leaders) and violate core societal values, mores, laws, or standards of proper conduct” (Umphress and Bingham, 2011, p. 622). UPB thus seems to be beneficial to the organization in the short term but can be harmful to the organization and society in the long term. Exploring the causes of UPB is necessary to prevent and control it (Umphress et al., 2010; Chen et al., 2021). As research on UPB causes has intensified, the idea that UPB is a stress-coping strategy for employees has received attention (Thau et al., 2015; Zhang et al., 2018; Chen and Chen, 2021; Guo and Chen, 2021). Job insecurity, as “the sense of powerlessness to maintain desired continuity in a threatened job situation” (Greenhalgh and Rosenblatt, 1984, p. 438), is a common source of employee stress in today’s society. However, thus far, limited studies have been conducted on the relationship between job insecurity and UPB (Ghosh, 2017; Zhang et al., 2021): (1) Sufficient discussions on the internal mechanism of job insecurity affecting UPB are lacking, and the “black box” between them must be further opened. (2) Job insecurity is a two-dimensional concept, including quantitative (relating to the loss of job itself) and qualitative job insecurity (relating to the loss of valuable job characteristics) (Hellgren et al., 1999). These two dimensions emphasize different aspects of job insecurity, and some differences exist in their effects on individuals correspondingly (Hellgren et al., 1999; Reisel and Banai, 2002). For example, quantitative job insecurity is more related to individual stress symptoms than qualitative job insecurity (Tu et al., 2019). However, the effect of UPB by quantitative and qualitative job insecurity has not been investigated in separate, particularly comparative analysis on both, which limits the understanding of the causes of UPB. Therefore, our study explores the impact of quantitative and qualitative job insecurity on employees’ UPB on the basis of Chinese organizational situations, so as to address the above challenges and then provide new ideas for the management of UPB.

Conservation of resources (COR) theory, a fundamental motivational framework for research on individual responses in stressful situations, assumes that retaining, protecting, and building resources are a fundamental value pursuit for individuals (Hobfoll, 1989, 2011). Job security is commonly viewed as a valuable resource for individuals (Vásquez et al., 2020). The loss of a job (i.e., quantitative job insecurity) or the loss of valuable job characteristics such as opportunities for advancement (i.e., qualitative job insecurity) reflects that one is threatened by resource losses and is seen as a job stressor for individuals (Sverke et al., 2002; Rodriguez-Muñoz et al., 2012). Based on COR, individuals in the dilemma of resource loss threat have the motivation to protect and build resources (Hobfoll, 2011). In organizations, leaders control employees’ access to resources, and controlling their own impression in leaders’ minds is an important way for employees to protect and nurture their resources (Wang and Zhang, 2012; Zhang et al., 2018). That is, quantitative and qualitative job insecurity can stimulate employees’ impression management motivation, which is defined as “the degree to which people are motivated to control how others see them” (Leary and Kowalski, 1990, p. 34). Employees driven by impression management motivation adopt behaviors that can contribute to their organization, such as voice behavior (Choi et al., 2015) and organizational citizenship behavior (Grant and Mayer, 2009). As a behavior that can promote the effective operation of an organization or the effective work of its members, whether UPB is an impression management strategy of individuals has not been verified by scholars. Hence, our study attempts to reveal the relationships between quantitative, qualitative job insecurity and employees’ UPB from the impression management perspective. Furthermore, losing work is a wider range of resource losses than losing valuable work characteristics (Callea et al., 2019). Based on COR, individuals faced with quantitative and qualitative job insecurity have different motivation strengths to control leaders’ perceptions of them in response to the resource loss threat of varying intensities (Greenhalgh and Rosenblatt, 1984; Hobfoll et al., 2018). The likelihood of individuals subsequently adopting UPB is also different. To this end, our study determines whether differences exist in the degrees of correlations between quantitative, qualitative job insecurity, and UPB from the impression management motivation perspective.

In real life, not all individuals who generate impression management motivation adopt UPB to attempt to manage their impressions in the minds of others. The two-component model of impression management states that impression management is a two-stage process, including impression management motivation and impression construction (Leary and Kowalski, 1990). When individuals with impression management motivation choose specific manners to carry out impression management, they are influenced by factors such as individual self-concept and desired/undesired identity (Leary and Kowalski, 1990). To this end, we choose one situational variable strongly related to individual self-concept, namely, organizational identification, defined as “a perceived oneness with an organization and the experience of its successes and failures as one’s own” (Mael and Ashforth, 1992). Doing so helps us examine its moderating role in the relationship between impression management motivation and UPB. Employees with high organizational identification tend to construct their self-concept on the basis of organizational characteristics (Ravasi and Canato, 2013), act in accordance with organizational values (Scholl et al., 2018), and even break through social moral standards to maintain organizational interests (Chen et al., 2016, 2020). Individuals with high organizational identification who generate impression management motivation are sensitive to the “pro-organizational” characteristics of UPB, having a high probability of adopting this behavior. By contrast, the self-concept construction of employees with low organizational identification is not dependent on their organization (Ravasi and Canato, 2013). For this reason, striving to maintain and improve their position in their organization is not their only strategy for dealing with job insecurity. Even if they want to perform impression management to try to deal with quantitative and qualitative job insecurity, they choose conservative strategies and do not adopt UPB that is “immoral” to achieve organizational and leadership expectations. Once such a behavior is exposed, it will jeopardize its image outside the organization. Therefore, this article speculates that organizational identification moderates the mediating role of impression management motivation in the relationships between quantitative, qualitative job insecurity, and UPB.

In brief, our study develops a research model on the basis of COR and the dual-component model of impression management, with impression management motivation as a mediating variable and organizational identification as a moderating variable, to explain employees’ UPB under the influence of quantitative and qualitative job insecurity. In so doing, this study makes several contributions. First, we simultaneously focus on the two dimensions of job insecurity—qualitative and quantitative—and comparatively investigate their relationship with employees’ UPB, responding to the call for “the need for comparative studies of quantitative and qualitative job insecurity” (Sverke et al., 2002; Tu et al., 2019) and enriching job insecurity literature. Second, we introduce a new mediating factor (i.e., impression management motivation) to explain the relationships between quantitative, qualitative job insecurity, and UPB, providing a new theoretical perspective for determining UPB causes. Last, we investigate the moderating role of organizational identification in the process of job insecurity affecting UPB, which further uncovers the “dark side” of organizational identification and provides new evidence for enterprise management practices.

## Theory background and hypotheses

Conservation of resources is an important motivational framework for understanding individual behaviors under stressful events proposed by Hobfoll (1989). COR begins with the tenet that individuals strive to obtain, retain, foster, and protect those things they centrally value. This core tenet follows a number of principles, two of which are very important (Hobfoll, 1989, 2001, 2011; Hobfoll et al., 2018). The first principle is that resource loss is disproportionately more salient than resource returns. And the second principle is that people must invest resources in order to prevent resource loss, recover from losses, and gain resources. These two principles of COR help explain the process mechanisms of quantitative and qualitative job insecurity affects UPB. According to the first principle, stable work and high-quality employment relationships are resources that employees value. When employees face quantitative and qualitative job insecurity, the potential loss of resources threatens them. Moreover, because unemployment is a wider loss than the loss of some important job characteristics, individuals generally perceive the threat of resource loss more strongly when faced with quantitative job insecurity than when faced with qualitative job insecurity (Hellgren et al., 1999; Callea et al., 2019). According to the second principle, individuals threatened by resource loss will be motivated to invest in resources and the intensity of motivation will be affected by the magnitude of the threat of resource loss. In an organization, leaders control employees’ access to resources, and establishing their desired image in leaders’ minds is an important way for employees to invest in resources. The motivation to control one’s own impression in the minds of others, known as impression management motivation, is an important part of the two-component model of impression management (Leary and Kowalski, 1990).

The two-component model of Impression management fully describes the processes involved in the behavior related to impression, including the two discrete stages of impression management motivation and impression (Leary and Kowalski, 1990). When individuals who generate impression management motivation engage in impression construction, that is, when they choose specific strategies to manage impression, they will be influenced by situational factors, such as self-concept, desired and undesired identities (Leary and Kowalski, 1990). Organizational identification is a concept strongly related to self-concept (Mael and Ashforth, 1992). Thus, this study uses organizational identification as a moderator variable to understand whether individuals with impression management motivation due to quantitative and qualitative job insecurity will adopt UPB as an impression management strategy.

**FIGURE 1:**
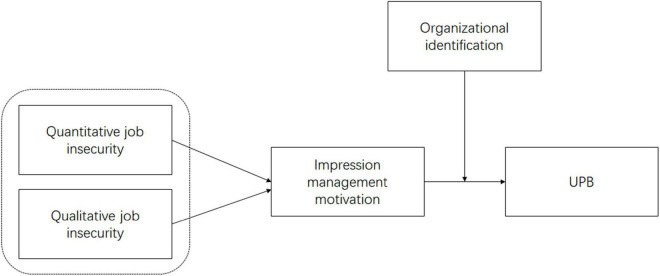
Theoretical model.

To this end, based on COR and two-component model of impression management, we demonstrate the impact of quantitative and qualitative job insecurity on employees’ UPB in the Chinese context, with impression management motivation as a mediating variable and organizational identification as a moderating variable. Figure 1 is the theoretical model of our study, which we will describe in detail below.

### Job insecurity and unethical pro-organizational behavior

Unethical pro-organizational behavior refers to “actions that are intended to promote the effective functioning of the organization or its members (e.g., leaders) and violate core societal values, mores, laws, or standards of proper conduct” (Umphress and Bingham, 2011, p. 622), such as falsifying financial data to drive up stock prices and deliberately concealing product defects to increase product sales. Such a behavior has two conflicting attributes: pro-organizational and unethical (Umphress et al., 2010). The pro-organizational nature of UPB temporarily benefits recipients, but its unethical nature can ultimately divert it from its original intent and cause a series of harms, jeopardizing the sustainable development of an organization and the interests of other stakeholders (Umphress et al., 2010; Lee et al., 2019). In view of the harms caused by the unethical nature of UPB, scholars have devoted themselves to exploring its causes to provide theoretical guidance for the prevention and control of this behavior. With in-depth research on UPB causes, the view that UPB is an employee stress-coping strategy has received attention (Thau et al., 2015; Zhang et al., 2018; Chen and Chen, 2021; Guo and Chen, 2021). Among them, job insecurity is seen as a common stressor, and its impact on individual UPB must be further revealed.

Job insecurity refers to “a sense of powerlessness to maintain desired continuity in a threatened job situation” (Greenhalgh and Rosenblatt, 1984, p. 438), which can span the range from threats of imminent job loss to loss of important job features (e.g., promotion space, salary development, job autonomy) (Greenhalgh and Rosenblatt, 1984), i.e., job insecurity includes quantitative and qualitative job insecurity (Hellgren et al., 1999). Both are recognized as job stressors, harming employees’ health, mood, and wellbeing and affecting their job performance (Ferrie et al., 2001; De Witte et al., 2010, 2016). In addition, some scholars have pointed out that quantitative and qualitative job insecurity emphasize different aspects of job insecurity, some differences exist in the extent to which they affect outcomes (e.g., job involvement, stress symptoms) (Xiao et al., 2018; Tu et al., 2019). However, only a limited number of studies have examined the comparative consequences of both types (Long et al., 2022). To this end, we respond to the call of scholars such as Tu et al. (2019) to conduct separate research and a comparative analysis on the impact of quantitative and qualitative job insecurity on employees’ UPB in organizations.

Conservation of resources is often used to explain individual behavioral choices in stressful situations, with the central idea that individuals strive to obtain, retain, protect, and foster the things they value. These valued entities are termed resources and may be delineated into object, condition, personal characteristic, and energy resources (Hobfoll, 1989; Hobfoll et al., 2018). Among them, continuous (the subject of quantitative job insecurity) and high-quality (the subject of qualitative job insecurity) employment relationships are the important resources that employees aim to preserve (Vásquez et al., 2020). As serious job stressors, quantitative and qualitative job insecurity pose a threat to the resources that employees value, thus motivating them to monitor specific threatening stimuli, such as possible organizational downsizing actions or organizational change activities (Låstad et al., 2015). However, this constant attention to environmental uncertainty consumes individuals’ psychological energy, leading to a further increase in individual job insecurity (Sverke et al., 2002; Charkhabi, 2019). In terms of COR theory, individuals whose resources are threatened while protecting and maintaining their existing resources want to acquire external resources to increase their resource stock or to replenish the energy that has been lost (Hobfoll, 2001). Specifically, given that the threat has not yet finally emerged, individuals who concern about the continued existence of their job (quantitative insecurity) and important job features (qualitative insecurity) try to suppress bad feelings and work hard to demonstrate their value to the organization they belong to Otto et al. (2011), Hewlin et al. (2016), Shoss (2017). UPB is pro-organizational in nature. Individuals can use this behavior to achieve the desired results of their organization and thus show self-worth. UPB is likely to become a resource conservation means for employees under the influence of the two types of job insecurity.

Although both job loss and loss of certain important job characteristics involve potential resource loss, the perceived threat of resource loss by employees can vary (Reisel and Banai, 2002). This is because losing a job usually means losing all its valuable characteristics, including compensation and promotion prospects, opportunities for training and development, interpersonal relationships within the organization, etc (De Witte et al., 2010; Callea et al., 2019). That is, compared with qualitative job insecurity, individuals facing quantitative job insecurity would perceive a stronger resource loss threat (Hellgren et al., 1999), and they are more motivated to change the bad situation they find themselves in. These individuals are also sensitive to the potential benefits of adopting UPB, such as contributing to their organization and impressing others that they are valuable to their organization, and are likely to adopt it. On the basis of the above inferences, the following hypotheses are proposed:

***Hypothesis 1 (H1):*** Quantitative (a) and qualitative (b) job insecurity are positively related to UPB.

***Hypothesis 2 (H2):*** Quantitative job insecurity is more strongly positively related to UPB than qualitative job insecurity.

### Mediating role of impression management motivation

Impression management, the process by which people control the impressions others form of them, plays an important role in interpersonal behavior (Bolino et al., 2016). The two-component model of impression management conceptualizes impression management as being composed of two discrete processes: impression management motivation and impression construction (Leary and Kowalski, 1990). Impression management motivation refers to “the degree to which people are motivated to control how others see them”; impression construction involves “the processes of determining the kind of impression one will try to make and choosing how one will go about making that impression” (Leary and Kowalski, 1990, p. 34).

The two-component model of impression management provides a comprehensive account of the processes involved in the impression-relevant behavior (Ginis and Leary, 2004). In this process, impression management motivation has a fundamental role and is mainly influenced by factors such as goal relevance, desired goal value, and discrepancy between desired and current image (Leary and Kowalski, 1990). For most people, maintaining the desired continuity in a work environment is an important goal in life (He et al., 2022). The sense of resource loss threat accompanies when individuals feel uncertain and powerless about maintaining the continuity of their expectations in an organizational setting (Sverke et al., 2002). COR states that when individuals are in situations where they have lost or are about to lose their resources, they not only develop the idea of preventing resource losses but also want to increase their resource stock to cope with present or future adverse situations (Hobfoll, 2011). In organizations where leaders control employee access to resources (Wang and Zhang, 2012; Zhang et al., 2018), constructing a self-image that is consistent with leadership expectations is an important way for employees to protect and nurture their resources (Choi et al., 2015; Klotz et al., 2018). Employees who face the potential threat of losing their jobs or losing valuable job characteristics are motivated to manage their image in line with their leaders’ expectations. That is, quantitative and qualitative job insecurity are positively correlated with employee impression management motivation, and this relationship has been confirmed (Huang et al., 2013).

According to impression management literature, the degree to which people control how others perceive them is influenced by the value and importance of desired goals (Leary and Kowalski, 1990; Bolino et al., 2016). Considering that the “iron rice bowl” (lifetime employment) concept has been dominant in Chinese society for a period, employees in Chinese companies have high job stability expectations (Tu et al., 2019). The loss of resources from a job loss is a greater threat to an employee than the loss of some important job characteristics, such as an opportunity for advancement (Greenhalgh and Rosenblatt, 1984; Tu et al., 2019). After all, the loss of certain important job characteristics indicates that a person still has a job to support a normal life. However, losing a job shows that a person can lose everything related to his job, including income, status, and opportunity for advancement (De Witte et al., 2010; Urbanaviciute et al., 2021). Based on COR, we suggest that being recognized by leaders and other organizational members is more valuable to employees facing quantitative job insecurity than facing qualitative job insecurity. That is, compared with qualitative job insecurity, quantitative job insecurity stimulates individuals to a high degree of motivation for impression management. Thus, the following hypotheses are proposed:

***Hypothesis 3 (H3):*** Quantitative (a) and qualitative (b) job insecurity are positively related to impression management motivation.

***Hypothesis 4 (H4):*** Quantitative job insecurity is more strongly positively related to impression management motivation than qualitative job insecurity.

Given that a person is motivated to create an impression on others, the issue is determining precisely the kind of impression one wants to make and choosing how one will go about making that impression (Leary and Kowalski, 1990). Making contributions to organizations is what all leaders expect from their employees (Qu et al., 2016). Studies have found that individuals with impression management motivation take organizational citizenship behavior (Grant and Mayer, 2009), voice behavior (Choi et al., 2015), and other behaviors that can make contributions to organizations (Bolino et al., 2016). UPB, as a behavior that can promote the effective operation of an organization or the effective work of internal organization members, which is often acquiesced or even hinted by leaders (Zhang et al., 2018, 2021), is likely a means for employees to manage impressions. To sum up, this study suggests that individuals facing quantitative and qualitative job insecurity adopt UPB to engage in impression construction. Accordingly, the following hypothesis is proposed.

***Hypothesis 5 (H5):*** Impression management motivation mediates between quantitative (a), qualitative (b) job insecurity, and UPB.

### Moderating role of organizational identification

The two-component model of impression management states that impression management is a highly context-dependent phenomenon, and individuals who generate impression management motivation are influenced by factors such as self-concept and desired/undesired identity when choosing specific impression management strategies (Leary and Kowalski, 1990; Lee et al., 2020). Organizational identification refers to “a perceived oneness with an organization and the experience of the organization’s successes and failures as one’s own” (Mael and Ashforth, 1992, p. 103). It reflects the extent to which individuals add organizational characteristics to their self-concept (Li and Zhang, 2020) and is an important contextual factor that influences their behavior (Scholl et al., 2018). This study argues that organizational identification plays a moderating role in the process of quantitative and qualitative job insecurity affecting individual UPB through impression management motivation.

Impression management literature suggests that people value certain aspects of themselves that they will proudly display to others at appropriate times (Leary and Kowalski, 1990; Lee et al., 2020). Individuals with high organizational identification tend to define their self-concept through organization membership (Mael and Ashforth, 1992) and attach great importance to their such membership in the current organization (Marstand et al., 2021). Therefore, they take the initiative to consider problems from the organizational perspective (Scholl et al., 2018) and even break through the social moral code to realize organization interests (Chen et al., 2016, 2020; Conroy et al., 2017). When individuals with high organizational identification are driven by impression management motivation, they pay attention to the “pro-organization” characteristics of UPB. Contributing to their organization can also help them consolidate their membership, and employees are highly likely to adopt UPB for impression construction, which can further assist them in such consolidation. The possibility of adopting UPB is also high. By contrast, the positive self-image desired by low organizational identification employees is not dependent on organizational membership (Lee et al., 2020), and their attachment and loyalty to their organization are low. Even if they want to change leaders’ impressions of them by contributing to organizations, they do not attempt UPB that may damage their external professional reputation. Accordingly, the following hypothesis is proposed.

***Hypothesis 6 (H6):*** Organizational identification moderates the positive effect of impression management motivation on UPB, such that the effect is strong when organizational identification is high, rather than low.

According to the above hypothetical logic of mediating and moderating effects, for individuals with high organizational identification, being forced to leave their organization or having limited development prospects in their organization not only threatens their subsistence income and status but also their self-value brought by organization membership (Crocker and Wolfe, 2001; Conroy et al., 2017). As a result, they are threatened with very serious resource losses (Schmitt and Branscombe, 2001). The resource investment principle of COR highlights the importance of proactive-coping COR theory, which suggests that under the resource loss threat, individuals are motivated to change their own behaviors to cope (Hobfoll, 2001; Schmitt and Branscombe, 2001), such as lowering their posture and increasing their contribution to their organization (Hobfoll et al., 2018). Employees with high organizational identification who face quantitative and qualitative job insecurity are sensitive to UPB’s function of “showing loyalty to an organization and obedience to the leadership of the organization” (Zhang et al., 2018). These employees also choose to adopt this behavior to deal with the strong resource loss threat. On the contrary, employees with low organizational identification have a weak sense of belonging and low commitment to their organization (Glavas and Godwin, 2013). Perceiving resource losses in the face of significant job features, which are about to be lost or even existing jobs, is difficult for such employees. For them, their self-esteem is not based on their current work achievements (Mael and Ashforth, 1992), and maintaining and improving their position is not their only strategy to deal with job insecurity; individuals may choose to quit or even fight against it (Shoss, 2017). Even if individuals with low organizational identification want to carry out impression management to attempt to deal with quantitative and qualitative job insecurity, they do not adopt UPB that may harm their career development outside their organization to meet organizational and leadership expectations. In summary, the following hypothesis is proposed:

***Hypothesis 7 (H7):*** Organizational identification moderates the mediating role of impression management motivation in the relationship between quantitative (a), qualitative (b) job insecurity, and UPB, such that the effect is strong when organizational identification is high, rather than low.

## Materials and methods

### Participants and procedures

Questionnaires were used to survey full-time employees of five enterprises in China. These enterprises cover a range of industries, including insurance, finance, real estate, and service.

To strengthen the evidence supporting the proposed relationships among variables under investigation, a multiphase procedure was used to conduct the survey in two phases. Each of the five enterprises had a contact person who delivered surveys during each phase. In the first phase, data were obtained for employees’ perceived job insecurity and respondents’ demographic information. One month later, the second phase was initiated. This phase measured respondents’ impression management motivation, UPB, and organizational identification. A coding scheme was used to match surveys from the two phases and yet ensure anonymity of the respondents.

A total of 350 employees were randomly selected to participate in the survey. The elimination of invalid questionnaires (failed to complete all two phases) resulted in 254 completed surveys (72.571% completion rate). Demographic information showed that 55.118% of samples were female, 93.701% were aged 20–39, 79.921% were bachelor degree and above, 81.102% were general employees, and 75.197% positional tenure with their current organization were for 0–10 years.

### Measures

Given that this study was conducted in China but all scales used were originally written in English, translation and back-translation were performed in a manner consistent with established cross-cultural translation procedures. Except for demographic variables, other variables were measured using a five-point Likert scale scoring method.

Job insecurity was measured using a two-dimensional scale developed by Hellgren et al. (1999), with quantitative job insecurity containing three items, such as “As things stand now, I am likely to lose my current job,” and qualitative job insecurity containing four items, such as “I will have some better opportunities for development in the company in the future.” The Cronbach’s alpha values for the quantitative and qualitative job insecurity scales were 0.838 and 0.860, respectively, suggesting that both scales had good reliability.

The impression management motivation drew on the five-dimensional, 22-item scale developed by Bolino and Turnley (1999). It also referred to previous research and the needs of this study to select two subscales, self-improvement and pandering, to form the impression management motivation questionnaire, with statements such as “I am willing to use flattery and favors to make leaders and colleagues like me more.” The Cronbach’s alpha value of the scale was 0.909, indicating that the scale had good reliability.

Organizational identification was measured using a six-item scale developed by Mael and Ashforth (1992), with statements such as “When someone criticizes my organization, I feel that they are criticizing me.” The Cronbach’s alpha value of the scale was 0.869, suggesting that the scale had good reliability.

A six-item scale developed by Umphress et al. (2010) was used for UPB, with items such as “If my organization needed me to, then I would withhold issuing a refund to a customer or client accidentally overcharged.” The Cronbach’s alpha value of the scale was 0.894, indicating that the scale had good reliability.

Based on studies related to UPB and previous research experience, gender, age, job position, and positional tenure were selected as control variables in our study.

### Data analysis methods

SPSS23.0 and AMOS23.0 were used for statistical analysis. First, confirmatory factor analysis (CFA) was conducted to evaluate the discriminant validity of each latent variable by using AMOS23.0. Second, SPSS23.0 was used for the descriptive statistical analysis and correlation analysis of variables. Finally, both hierarchical regression analysis by using SPSS23.0 and bootstrap by using PROCESS SPSS macro were employed to investigate the relationships among quantitative (qualitative) job insecurity, impression management motivation, and UPB, including the moderating role of organizational identification.

## Empirical analysis and research results

### Common method bias testing

To minimize common method bias (CMB), questionnaires were distributed and collected at two time points in this study, and they were filled out anonymously. However, CMB in the data may exist because all questions in the questionnaire are filled in by only one person (Podsakoff et al., 2012). For this reason, the CMB test was performed on the sample data before the hypothesis test. In this study, the Harman single-factor test method was adopted and SPSS 23.0 was used for the principal component factor analysis of all questionnaire items. The test results revealed that one single factor accounted for 25.122% of the variance, which is below 50%, thereby indicating no major CMB issues.

### Descriptive statistical analysis

The means, standard deviations (SDs), and correlation coefficients of all variables involved in this study are presented in Table 1. Quantitative job insecurity was significantly and positively correlated with impression management motivation (*r* = 0.345, *p* < 0.01); quantitative job insecurity was significantly and positively correlated with UPB (*r* = 0.380, *p* < 0.01); qualitative job insecurity was significantly and positively correlated with impression management motivation (*r* = 0.220, *p* < 0.01); qualitative job insecurity and UPB positive correlation was insignificant (*r* = 0.123, *p* > 0.05); impression management motivation was significantly positively correlated with UPB (*r* = 0.259, *p* < 0.01). Except for the correlation between qualitative job insecurity and UPB, which was unverified, the correlation analysis results of other variables tentatively verified our research hypotheses.

**TABLE 1:** Variable means, standard deviations (SDs), and correlation coefficients (*N* = 254).

Variable	1	2	3	4	5	6	7	8	9
(1) Gender	−								
(2) Age	–0.102	−							
(3) Position	−0.257**	0.537**	−						
(4) Positional tenure	0.000	0.781**	0.431**	−					
(5) QUAN	0.014	0.056	–0.079	0.027	−				
(6) QUAL	–0.122	–0.066	0.047	−0.147*	0.097	−			
(7) IMM	0.049	–0.066	0.011	–0.112	0.345**	0.220**	−		
(8) OI	–0.023	0.035	0.046	–0.114	0.329**	0.149*	0.245**	−	
(9) UPB	0.002	–0.025	–0.030	–0.066	0.380**	0.123	0.259**	0.401**	−
*Mean*	1.550	2.250	1.540	1.360	2.038	3.482	3.028	2.898	2.371
*SD*	0.498	0.562	0.832	0.868	0.982	0.817	0.856	0.817	0.916

QUAN, quantitative job insecurity; QUAL, qualitative job insecurity; IMM, impression management motivation; OI, organizational identification.

**p < 0.01, *p < 0.05.

### Discriminant validity analysis

We conducted CFA to assess the discriminant validity of the key variables using AMOS23.0. As shown in Table 2, the fit indicators of the five-factor model all met the accepted standards (χ^2^/*df* = 2.593 < 3, *CFI* = 0.915, *TLI* = 0.902, *RMSEA* = 0.076, and *SRMR* = 0.063) and were better than other alternative models. Therefore, the five variables had good discriminant validity and could be tested in the next step.

**TABLE 2:** Results of confirmatory factor analysis (CFA) (*N* = 254).

Model	χ^2^	*df*	χ^2^/*df*	*CFI*	*TLI*	*RMSEA*	*SRMR*
Five-factor (OI, QUAN, QUAL, IMM, UPB)	951.650	367	2.593	0.915	0.902	0.076	0.063
Four-factor (OI + QUAN, QUAL, IMM, UPB)	1218.609	371	3.285	0.855	0.837	0.082	0.084
Three-factor (OI + QUAN + QUAL, IMM, UPB)	1570.607	374	4.199	0.696	0.672	0.113	0.108
Two-factor (OI + QUAN + QUAL + IMM, UPB)	2160.867	376	5.747	0.525	0.487	0.142	0.144
One-factor (OI + QUAN + QUAL + IMM + UPB)	2819.929	377	7.480	0.381	0.335	0.160	0.152

“+” indicates that the two factors are combined.

### Testing for main and mediating effects

We conducted hierarchical regression analyses to test our hypotheses, and the analysis results are presented in Table 3.

**TABLE 3:** Results of the multiple regression analysis of mediating effects (*N* = 254).

Variable	IMM			UPB					
	Model 1	Model 2	Model 3	Model 4	Model 5	Model 6	Model 7	Model 8	Model 9
**Control variables**									
Gender	0.129	0.135	0.162	0.009	0.016	0.029	–0.026	–0.003	–0.013
Age	0.041	–0.043	0.026	0.118	0.022	0.109	0.107	0.029	0.102
Position	0.105	0.171	0.082	–0.026	0.049	–0.040	–0.055	0.025	–0.061
Positional tenure	–0.168	–0.155	–0.122	–0.124	–0.109	–0.096	–0.077	–0.086	–0.064
**Independent variables**									
QUAN		0.315***			0.359***			0.313***	
QUAL			0.221**			0.132			0.074
**Mediating variables**									
IMM							0.275***	0.145*	0.260***
*R* ^2^	0.022	0.150	0.065	0.007	0.151	0.020	0.071	0.167	0.075
Δ*R*^2^	0.006	0.133	0.046	–0.009	0.134	0.000	0.052	0.147	0.052
*F*	1.412	8.746***	3.435**	0.408	8.849***	0.998	3.786**	8.249***	3.335**

***p < 0.001, **p < 0.01, *p < 0.05.

Test of the relationship between quantitative, qualitative job insecurity, and UPB. Model 5 indicates that the effect of quantitative job insecurity on UPB was significant (*r* = 0.359, *p* < 0.001), and H1a was verified. Model 6 shows that the effect of qualitative job insecurity on UPB was insignificant (*r* = 0.132, *p* > 0.05), and H1b was unverified. To further compare the relative strength of the relationship of quantitative and qualitative job insecurity with employee outcome, we conducted dominance analysis (Azen and Budescu, 2003). This method is designed to evaluate the relative importance of correlated predictors and has been widely used in organizational research (Lebreton et al., 2004). We first computed the average increase in *R*^2^ for each predictor across all possible subset regression models, and then divided the average increase in *R*^2^ for each predictor by the total variance explained in the outcome. Quantitative job insecurity accounted for 27.348% of the predictable criterion variance of UPB, compared with 7.260% explained by qualitative job insecurity. Therefore, H2 was supported.

Test of the relationship between quantitative, qualitative job insecurity, and impression management motivation. Model 2 reveals that the effect of quantitative job insecurity on impression management motivation was significant (*r* = 0.315, *p* < 0.001), and H3a was verified. Model 3 shows that the effect of qualitative job insecurity on impression management motivation was significant (*r* = 0.221, *p* < 0.01), and H3b was confirmed. To further compare the relative strength of the relationship of quantitative and qualitative job insecurity with impression management motivation, we performed dominance analysis (Azen and Budescu, 2003). We initially computed the average increase in *R*^2^ for each predictor across all possible subset regression models, and then divided the average increase in *R*^2^ for each predictor by the total variance explained in the outcome. Quantitative job insecurity accounted for 22.375% of the predictable criterion variance of impression management motivation, compared with the 13.885% explained by qualitative job insecurity. Thus, H4 was supported.

The mediating effect test was performed using the causal steps approach of Baron and Kenny (1986). In Model 2, quantitative job insecurity (independent variable) had a significant effect on UPB (dependent variable), satisfying the first condition of the mediating effect test. From Model 8, when quantitative job insecurity and impression management motivation entered the regression equation at the same time, the positive effect of impression management motivation on UPB was significant (*r* = 0.145, *p* < 0.05); the regression coefficient of quantitative job insecurity on UPB was also significant (*r* = 0.313, *p* < 0.001), confirming the second condition of the mediating effect test. Therefore, impression management motivation played a mediating role in the effect of quantitative job insecurity on UPB, and H5a was supported. From Model 9, when qualitative job insecurity and impression management motivation entered the regression equation, the positive effect of impression management motivation on UPB was significant (*r* = 0.260, *p* < 0.001). Thus, impression management motivation played a mediating role in the effect of qualitative job insecurity on UPB, and H5b was verified.

### Testing for moderating effects

**FIGURE 2:**
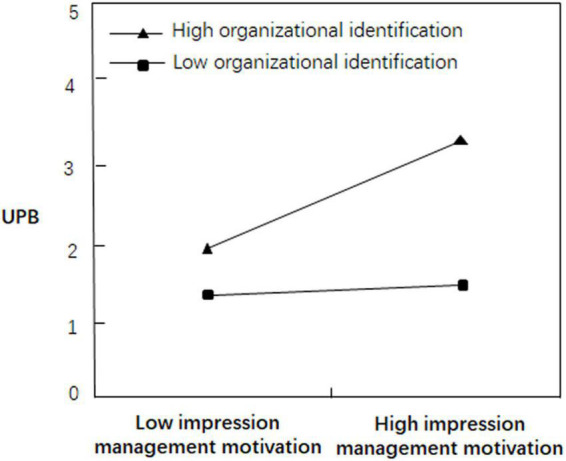
Moderating effect of organizational identification on the relationship between impression management motivation and UPB.

We used Baron and Kenny’s (1986) research procedure to examine the moderating effect of organizational identification, and the results are presented in Table 4. From Model 11, when the interaction effect terms of impression management motivation, organizational identification, and impression management motivation and organizational identification entered the regression equation simultaneously, the interaction effect term of impression management motivation and organizational identification on UPB was significant (*r* = 0.208, *p* < 0.01), indicating that organizational identification moderated the effect of impression management motivation on UPB. This finding supported H6. From Model 13, the effect of impression management motivation on UPB was significant (*r* = 0.137, *p* < 0.05). When the terms of quantitative job insecurity, impression management motivation, organizational identification and the interaction effect of impression management motivation and organizational identification entered the regression equation simultaneously, the interaction effect of impression management motivation and organizational identification on UPB also was significant (*r* = 0.184, *p* < 0.05), suggesting that organizational identification moderated the mediating effect of quantitative job insecurity on UPB, and H7a was supported. The same research procedure analysis revealed the mediating effect of organizational identification moderating qualitative job insecurity on UPB, and H7b was supported. To visually reflect the moderating effect of organizational identification, Figure 2 was plotted.

**TABLE 4:** Results of the multiple regression analysis of moderating effects (*N* = 254).

Variable	UPB					
	Model 10	Model 11	Model 12	Model 13	Model 14	Model 15
**Control variables**						
Gender	–0.023	–0.030	–0.008	–0.015	0.001	–0.013
Age	0.060	0.067	0.016	0.024	0.064	0.038
Position	–0.070	–0.073	–0.012	–0.018	–0.031	–0.048
Positional tenure	–0.049	–0.050	–0.061	–0.061	–0.085	–0.065
**Independent variables**						
QUAN			0.216***	0.206**		
QUAL					0.026	0.027
IMM	0.178**	0.208**	0.107	0.137*	0.170**	0.201**
**Moderating variable**						
OI	0.451***	0.442***	0.370***	0.366***	0.441***	0.437***
**Interaction variables**						
OI*IMM		0.211*		0.184*		0.214*
*R* ^2^	0.200	0.224	0.245	0.256	0.206	0.223
Δ*R*^2^	0.180	0.200	0.223	0.227	0.181	0.198
*F*	10.386***	10.046***	11.244***	10.698***	8.845***	8.859***

***p < 0.001, **p < 0.01, *p < 0.05.

On this basis, this study applied the PROCESS macro for SPSS developed by Hayes (2013) to validate the moderated mediation model as a whole. The results in Table 5 indicated that organizational identification positively moderated the mediating role of impression management motivation in the relationships between quantitative, qualitative job insecurity, and UPB. Specifically, when organizational identification was low, the indirect effect value of quantitative job insecurity on UPB *via* impression management motivation was 0.003, with a 95%CI of [−0.022, 0.046], containing “0,” which was insignificant; when organizational identification was high, the corresponding indirect effect value was 0.079, with a 95% CI is [0.019, 0.187], which was significant. When organizational identification was low, the indirect effect value of qualitative job insecurity on UPB *via* impression management motivation was 0.009, with a 95%CI of [−0.016, 0.052], containing “0,” which was insignificant; when organizational identification was high, the corresponding indirect effect value was 0.081, with a 95%CI of [0.024, 0.156], which was significant. The results confirmed H7a and H7b again.

**TABLE 5:** Mediating effects based on Bootstrapping at different levels of moderating variables (*N* = 254).

Variable	Item	Effect value	SE	Bias corrected 95% CI
QUAN	Lower OI (*Mean − SD*)	0.003	0.026	[−0.022, 0.046]
	Higher OI (*Mean* + *SD*)	0.079	0.032	[0.019, 0.187]
QUAL	Lower OI (*Mean − SD*)	0.009	0.016	[−0.016, 0.052]
	Higher OI (*Mean* + *SD*)	0.081	0.033	[0.024, 0.156]

## Discussion

In this study, based on COR and the dual-component model of impression management, a theoretical model with impression management motivation as the mediating variable and organizational identification as the moderating variable was constructed to explore how and when quantitative and qualitative affect UPB. Analysis based on 254 Chinese employee data shows that: (1) Quantitative job insecurity positively influences UPB, and the positive effect of qualitative job insecurity on UPB is insignificant. (2) Quantitative job insecurity positively affects impression management motivation and increases UPB. Although the direct effect of qualitative job insecurity on UPB is insignificant, it positively affects UPB through impression management motivation. (3) Organizational identification positively moderates the relationship between impression management motivation and UPB, as well as the indirect influence of quantitative and qualitative job insecurity on UPB through impression management motivation. Below, we discuss theoretical and practical implications, along with limitations and future directions of the present work.

### Theoretical implications

The theoretical contributions of this study are mainly reflected in the following points:

First, this work is a replication and extension of existing research on the relationship between job insecurity and employees’ UPB. Its findings, in a Chinese organizational context, support Ghosh’s (2017) argument that job insecurity is related to employees’ UPB. In response to the call of scholars to “conduct parallel studies on the effects of quantitative and qualitative job insecurity” (Sverke et al., 2002; Tu et al., 2019), our research performs a parallel investigation and comparison of the effects of the two types of job insecurity on UPB. Quantitative and qualitative job insecurity are revealed to have different effects on UPB, that is, the fear of losing the job itself is more likely to trigger individual UPB than the loss of important job characteristics. The discovery of the roles of the two-dimension differences not only deepens the understanding of the relationship between job insecurity and UPB but also increases existing literature about which job insecurity dimension, qualitative or quantitative, may lead to serious harmful effects (Tu et al., 2019), thereby enriching the existing theoretical system of job insecurity.

Second, although the influence of job insecurity on employees’ UPB has been initially explored (Ghosh, 2017; Lawrence and Kacmar, 2017), the “black box” between the two must be further opened. To fill the gap, on the basis of COR, our study verifies the mediating role of impression management motivation between quantitative, qualitative job insecurity, and UPB. Due to different resource loss threats, the motivation degree of individuals to control their image in leaders’ minds in the face of quantitative and qualitative job insecurity is different. The possibility of individuals adopting UPB is also different. The role of impression management motivation in the relationship between quantitative (qualitative) job insecurity and UPB is verified for the first time, which not only extends existing research on UPB motivation but also further opens the “black box” of quantitative and qualitative job insecurity on UPB, providing a new theoretical perspective for understanding UPB under the influence of both types of job insecurity.

Last, this study examines, for the first time, the moderating role of organizational identification in the process of quantitative and qualitative job insecurity acting on UPB. It finds that organizational identification strengthens the influence of impression management motivation on UPB and enhances the mediating role of impression management motivation between quantitative, qualitative job insecurity, and UPB. That is, whether individuals motivated by impression management due to quantitative and qualitative job insecurity adopt UPB as an impression management strategy depends, to a certain extent, on their organizational identification level. For employees with high organizational identification, being forced to leave their organization or lose job characteristics not only damages their conditional resources for survival and development but also damages their personal characteristic resources. They are sensitive to the “pro-organization” characteristic of UPB and are willing to adopt UPB for impression construction. Conversely, for employees with low organizational identification, their self-concept is not dependent on their organization, and the quantitative and qualitative job insecurity threat is small. They are sensitive to the “immoral” nature of UPB and are less likely to adopt UPB, which damages their image outside their organization, to meet the expectations of others within their organization. In the theoretical model, the exploration of the moderating role of organizational identification not only extends the boundary condition of the dual-component model of impression management but also helps researchers recognize the “dark side” of organizational identification (Conroy et al., 2017) in the job insecurity outcome.

### Practical implications

The managerial implications of this study are mainly as follows:

First, organizations should make efforts in identifying sources that may lead to job insecurity, especially during environmental instability periods, such as layoff and organizational restructuring periods. Organizations must also inform employees of major changes that may or may not occur or involve them in the decision-making process to reduce employee job insecurity and attenuate the negative effects of job insecurity.

Second, managers should accurately identify and reasonably respond to employees’ impression management strategies. In a highly unstable environment, managers should be wary of subordinates’ “sugar-coated” and excessive self-promotion, should see through appearances to objective substance, and objectively assess subordinates’ real abilities and performance levels to prevent individuals from taking UPB in response.

Finally, organizations should be aware that individuals with high organizational identification may adopt UPB, which can bring temporary benefits but has long-term risks, to protect work and maintain organizational membership when they are faced with uncertain factors. For this reason, organizations should not only socialize employees in training and culture-building activities but also guide them in establishing positive career views and correct work ethics. Moreover, organizations must help employees in reducing their negative reactions to job insecurity through a series of initiatives, which can enhance individual career development to achieve organizational flexibility.

### Limitations and prospects

Future research can address our study limitations. First, we conclude that job insecurity brings about UPB, which is a common phenomenon in business societies, but we only collect data from China. In the future, data from different countries can be obtained for performing cross-cultural research on UPB. Second, the direct effect of qualitative job insecurity on employees’ UPB is insignificant, but it affects employees’ UPB through the mediating effect of impression management motivation. We speculate that there may be other mediating mechanisms between the two that create a masking effect with the mediating mechanism of impression management motivation. Subsequent studies may further clarify the complex relationship between qualitative job insecurity and UPB from other theoretical perspectives. Finally, we only investigate the moderating effect of organizational identification in the process of job insecurity influencing employees’ UPB and failed to examine the roles of other individual-level variables, such as moral identification, and organizational-level factors, such as leadership behaviors and human resource management practices. Future research can consider the moderating effects of these factors and further refine the theoretical model.

## Conclusion

The relationship between job insecurity and UPB has been preliminarily explored (Ghosh, 2017; Zhang et al., 2021), but some issues related to this have not been fully addressed. Frist, the internal mechanism of job insecurity affecting UPB is rarely discussed, and the “black box” between them should be opened. Moreover, job insecurity is a two-dimensional concept, including quantitative and qualitative job insecurity, but there is a lack of comparative analysis of the effect of the two on UPB. To address gaps, our study exam the effect of quantitative and qualitative job insecurity on UPB by focusing on the mediating effect of impression management motivation and the moderating effect of organizational identification, based on COR and the two-component model of impression management. Our findings suggest that: (1) Quantitative job insecurity positively influences UPB to a higher degree than qualitative job insecurity. (2) Impression management motivation as a mediator links the relationship between quantitative, qualitative job insecurity, and UPB and explains why there are differences in the extent to which qualitative and quantitative job insecurity affects UPB. (3) Organizational identification, which moderates the mediating effect of impression management motivation upon the relationship between the two types of job insecurity and UPB, is a key boundary condition of quantitative, qualitative job insecurity influencing UPB. Our findings extend the understanding of the relationship between job insecurity and UPB, make several key theoretical contributions to the mechanism and the boundary condition of how UPB might occur, and contribute to practical implications.

## Data availability statement

The raw data supporting the conclusions of this article will be made available by the authors, without undue reservation.

## Author contributions

LX: research design, conception, original draft, data analysis, and critical revision of important intellectual content. TW: critical revision of important intellectual content, funding acquisition, and project administration. JW: critical revision of important intellectual content. All authors contributed to the article and approved the submitted version.
